# LncRNA GATA6-AS inhibits cancer cell proliferation and promotes cancer cell apoptosis in cervical cancer by down-regulating miR-205

**DOI:** 10.1186/s12905-020-01082-7

**Published:** 2020-11-09

**Authors:** Xiaoying Zhao, Huzhong Zheng, Jun Chen

**Affiliations:** 1 Department of Gynaecology and Obstetrics, Wenzhou Hospital Of Integrated Traditional China & Med, Wenzhou City, Zhejiang Province 325000 PR China; 2 Department of Immunology, Wenzhou Medical University, Chashan higher Education Park, Wenzhou City, Zhejiang Province 325000 PR China

**Keywords:** CSCC, GATA6-AS, miR-205, Proliferation, Apoptosis

## Abstract

**Background:**

Dysregulated endothelial cell growth is involved in many types of human cancer, including cervical cancer. LncRNA GATA6-AS was reported to regulate endothelial cell growth, suggesting it might involve in cervical cancer. Our study was carried out to explore the involvement of GATA6-AS in cervical squamous cell carcinoma (CSCC), a subtype of cervical cancer.

**Methods:**

To explore the expression of GATA6-AS, RT-qPCR was performed to detect GATA6-AS in plasma of 65 CSCC patients and 58 healthy females. To detect the expression of GATA6-AS, total RNAs were extracted.

**Results:**

We found that plasma GATA6-AS expression was down-regulated in CSCC patients than that in healthy females, and HPV infection did not significantly affect the plasma expression of GATA6-AS. Moreover, we found that plasma GATA6-AS showed diagnostic values for CSCC by performing ROC curve analysis. The expression of miR-205 in plasma was also found to be up-regulated in CSCC patients than that in healthy females and inversely correlated with the expression of GATA6-AS in CSCC patients. Furthermore, over-expression of miR-205 did not significantly affect the expression of GATA6-AS in CSCC cells, while over-expression of GATA6-AS down-regulated miR-205 expression. In addition, GATA6-AS over-expression inhibited CSCC cell proliferation and promoted CSCC cell apoptosis, while miR-205 over-expression played opposite roles and attenuated the effects of GATA6-AS over-expression on CSCC cells.

**Conclusion:**

Taken together, these results suggest that GATA6-AS may inhibit cell proliferation and promote cell apoptosis in CSCC by down-regulating miR-205.

## Background

As the second most common cancer in women, cervical cancer accounts for almost 12% of all female cancers [1, 2]. Each year, more than 500,000 cases of cervical cancer are reported worldwide, and more than 200,000 people die of it. At present, cervical cancer has become one of the main causes of cancer-related deaths [2]. Clinical studies have shown that human papillomaviruses (HPV) infection is related to more than 90% of cervical cancers [3]. HPV infection screening and HPV vaccination have significantly reduced the prevalence of HPV infection, leading to a decreased incidence of cervical cancer [4]. However, HPV-negative cervical cancer showed an increasing trend in recent years [5].

Besides HPV infection, genetic factors also contribute to the development and progression of cervical cancer [6, 7]. Analysis of human transcriptome has shown that only less than 2% of transcripts are related to protein-coding, and the rest are all non-coding RNAs (ncRNAs) [8]. There is accumulative evidence showing that different types of ncRNAs, such as long (> 200 nt) ncRNAs (lncRNAs), involve in diverse physiological and pathological processes, such as tumorigenesis [9, 10]. It has been well-established that certain lncRNAs in cancers, such as cervical cancer, regulate the expression of oncogenic and/or tumor suppressive genes to promote or inhibit cancer development [9, 10]. Therefore, regulation of lncRNA expression may assist cancer prevention and treatment [11]. However, the role of most lncRNAs in cancers remains unclear. It has been reported that GATA6-AS can regulate endothelial cell growth, which is known to be involve in the development of different types of human diseases, such as cervical cancer, by epigenetically regulating gene expression [12]. Our preliminary microarray data revealed that the expression of GATA6-AS down-regulated in cervical cancer, and its expression inversely correlated with miR-205 expression. In addition, it has also been showed that miR-205 play critical roles in many types of cancer [13–15]. Here, our study was carried out to investigate the involvement of GATA6-AS in cervical squamous cell carcinoma (CSCC), a major subtype of cervical cancers, and to explore its possible interaction with miR-205.

## Methods

### Research subjects

Our study included 65 patients with CSCC and 58 healthy females who were admitted by Wenzhou Medical University from May 2016 to May 2018. All CSCC patients were diagnosed by histopathological examinations. Among the 65 patients, 40 were HPV positive and 25 were HPV negative. Inclusion criteria of CSCC patients: 1) first time diagnosis; 2) no history of malignancy; 3) no therapy initiated before admission. Exclusion criteria: 1) with other clinical disorders besides CSCC; 2) patients transferred from other hospitals; 3) received any treatments within 3 months before admission. Age of the 65 patients with CSCC ranged from 30 to 61 years, with a mean age of 45.0 ± 4.2 year. Fifty-eight healthy females were enrolled in the physical health center of Wenzhou Medical University, and physical health parameters of them were all within normal range. Age of the 58 healthy females ranged from 32 to 64 years, and the mean age was 45.9 ± 4.3 year. This study was approved by Wenzhou Medical University Ethics Committee. All patients and healthy females signed informed consent.

### Specimens and cell lines

Fasting blood (5 ml) was extracted from each participant before therapies. Blood was centrifuged for 12 min at 1200 g in EDTA tubes to prepare plasma samples. C33A (HPV negative) and SiHa (HPV positive) cell lines were used in this study to perform in vitro experiments. These 2 cell lines were bought from ATCC (USA) and were cultivated in Eagle’s Minimum Essential Medium supplemented with 10% fetal bovine serum (FBS) in a 37 °C and 5% CO_2_ incubator.

### Real-time quantitative reverse transcription PCR (RT-PCR)

To detect the expression of GATA6-AS, total RNAs were extracted using Trizol reagent (Invitrogen, USA), SuperScript III Reverse Transcriptase (Thermo Fisher Scientific) was used to perform reverse transcriptions and Applied Biosystems™ PowerUp™ SYBR™ Green Master Mix was used to prepare all PCR reaction systems with 18S rRNA as endogenous control.

To detect the expression of miR-205, miRNAs were extracted using miRNeasy Kit (Qiagen). MiRNA reverse transcriptions were performed using TaqMan™ MicroRNA Reverse Transcription Kit (Applied Biosystems), and PCR reaction systems were prepared using TaqMan™ Fast Advanced Master Mix (Thermo Fisher Scientific). U6 was used as the endogenous control. 2^-∆∆CT^ method was used to perform all data normalizations.

### Transient transfection

Scrambled negative control miRNA and MISSION® microRNA Mimic hsa-miR-205 were purchased from Sigma-Aldrich (St. Louis, MO, USA). GATA6-AS full-length genomic DNA was inserted into the pcDNA3.1 vector to construct GATA6-AS expression vector. Lipofectamine 2000 reagent (Invitrogen, USA) was used to perform transient transfection with vectors and miRNAs at doses of 10 nM and 40 nM, respectively. Cells with no transfection were used as control cells. Cells transfected with negative control miRNA or empty vectors were used as negative control cells. Cells were collected at 24 h after transfection to perform subsequent proliferation and apoptosis assays.

### Cell proliferation assay

C33A and SiHa cells were collected at 24 h after transfection to perform in vitro cell proliferation assay. Eagle’s Minimum Essential Medium containing 10% FBS was used to prepare single-cell suspension and the cell concentration was 4 × 10^4^ cells/ml. After that, cells were cultivated in a 96-well plate with 0.1 ml cell suspension per well, followed by the addition of 10uL CCK-8 (Sigma-Aldrich) to each well every 24 h until 96 h. After that, cells were cultivated for additional 4 h, and 10 uL DMSO was added. After that, OD values (450 nm) were measured.

### Cell apoptosis assay

C33A and SiHa cells were collected at 24 h after transfection to perform in vitro cell apoptosis assay. Non-serum Eagle’s Minimum Essential Medium was used to prepare single-cell suspension, and the cell density was 6 × 10^4^ cells/ml. Cells were then cultivated in a 6-well plate with 2 ml per well. Cell culture was performed for 36 h and cells were then subjected to 0.25% trypsin digestion. Following staining with Annexin V-FITC and propidium iodide (PI), apoptotic cells were detected by flow cytometry.

### Statistical analysis

Each experiment was performed 3 times (biological replicates). Data were processed using GraphPad Prism 6 software. Comparisons between CSCC patients and healthy controls or between HPV-positive and HPV-negative patients were performed by unpaired t-test. Comparisons among different cell treatment groups were performed by ANOVA (one-way) and Tukey test. Linear regression was performed to analyze the correlation between plasma levels of miR-205 and GATA6-AS. Diagnostic values of plasma GATA6-AS for CSCC were evaluated by performing ROC curve analysis with CSCC patients as true positive cases and healthy females as true negative cases. Difference with *p* < 0.05 was considered as statistically significant.

## Results

### Plasma GATA6-AS expression was down-regulated in CSCC and not affected by HPV infection

**Fig. 1:**
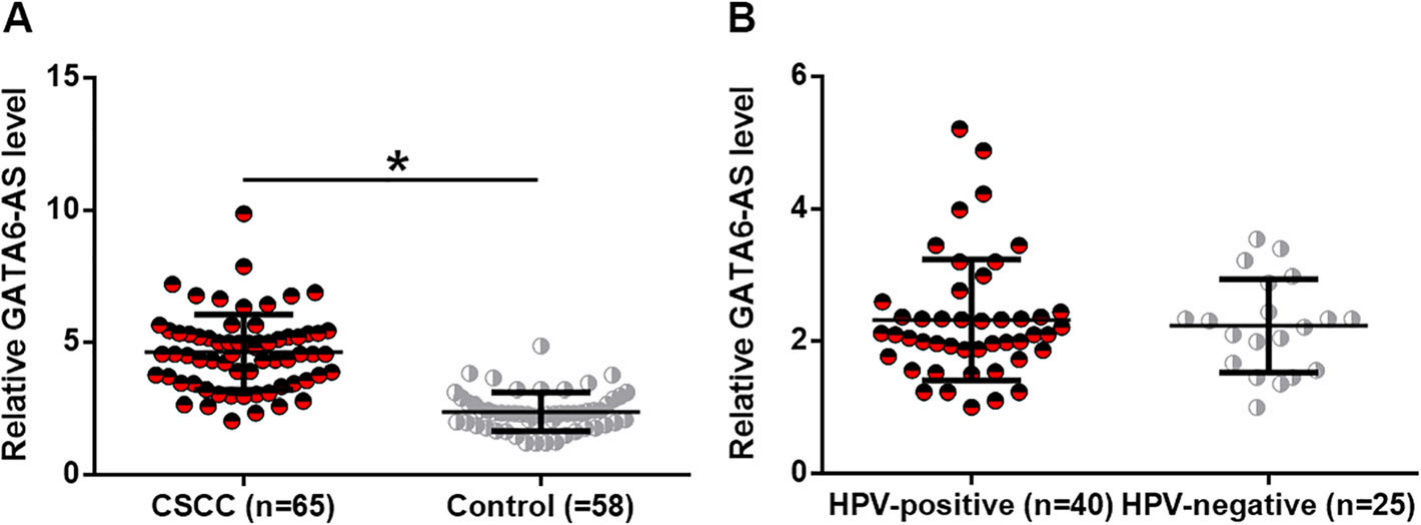
Plasma GATA6-AS was down-regulated in CSCC and not affected by HPV infection. RT-qPCR data showed that plasma GATA6-AS was down-regulated in CSCC patients than that in healthy females (**a**), (*, *p* < 0.05), and HPV infection did not significantly affect the plasma levels of GATA6-AS (**b**)

To explore the expression of GATA6-AS, RT-qPCR was performed to detect GATA6-AS in plasma of 65 CSCC patients and 58 healthy females. Compared with healthy females, the plasma levels of GATA6-AS were significantly lower in CSCC patients (Fig. 1a, *p* < 0.05). Among the 65 patients, 40 were HPV positive patients and 25 were HPV negative patients. As shown in Fig. 1b, no significant differences in plasma levels of GATA6-AS was found between HPV-positive and HPV-negative patients (*p* < 0.05).

### Plasma GATA6-AS showed diagnostic values for CSCC

**Fig. 2:**
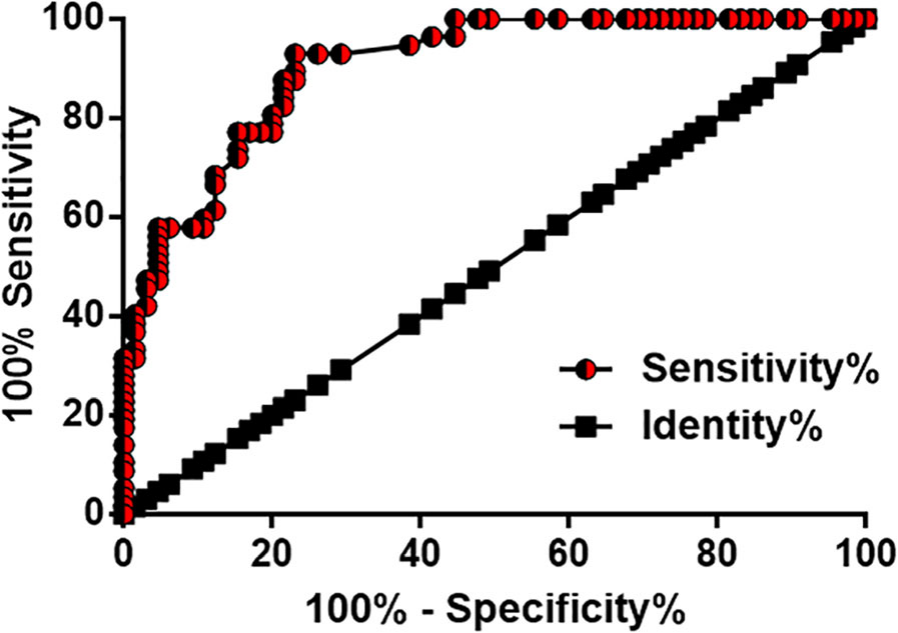
Plasma GATA6-AS has diagnostic values for CSCC. ROC curve analysis showed that down-regulation of GATA6-AS expression distinguished CSCC patients from healthy females

Diagnostic values of plasma GATA6-AS for CSCC were evaluated by performing ROC curve analysis with CSCC patients as true positive cases and healthy females as true negative cases. As shown in Fig. 2, the area under the curve was 0.9035, with a standard error of 0.02595 and 95% confidence interval of 0.8526–0.9544.

### Plasma miR-205 expression was upregulated in CSCC patients and inversely correlated with GATA6-AS expression

**Fig. 3:**
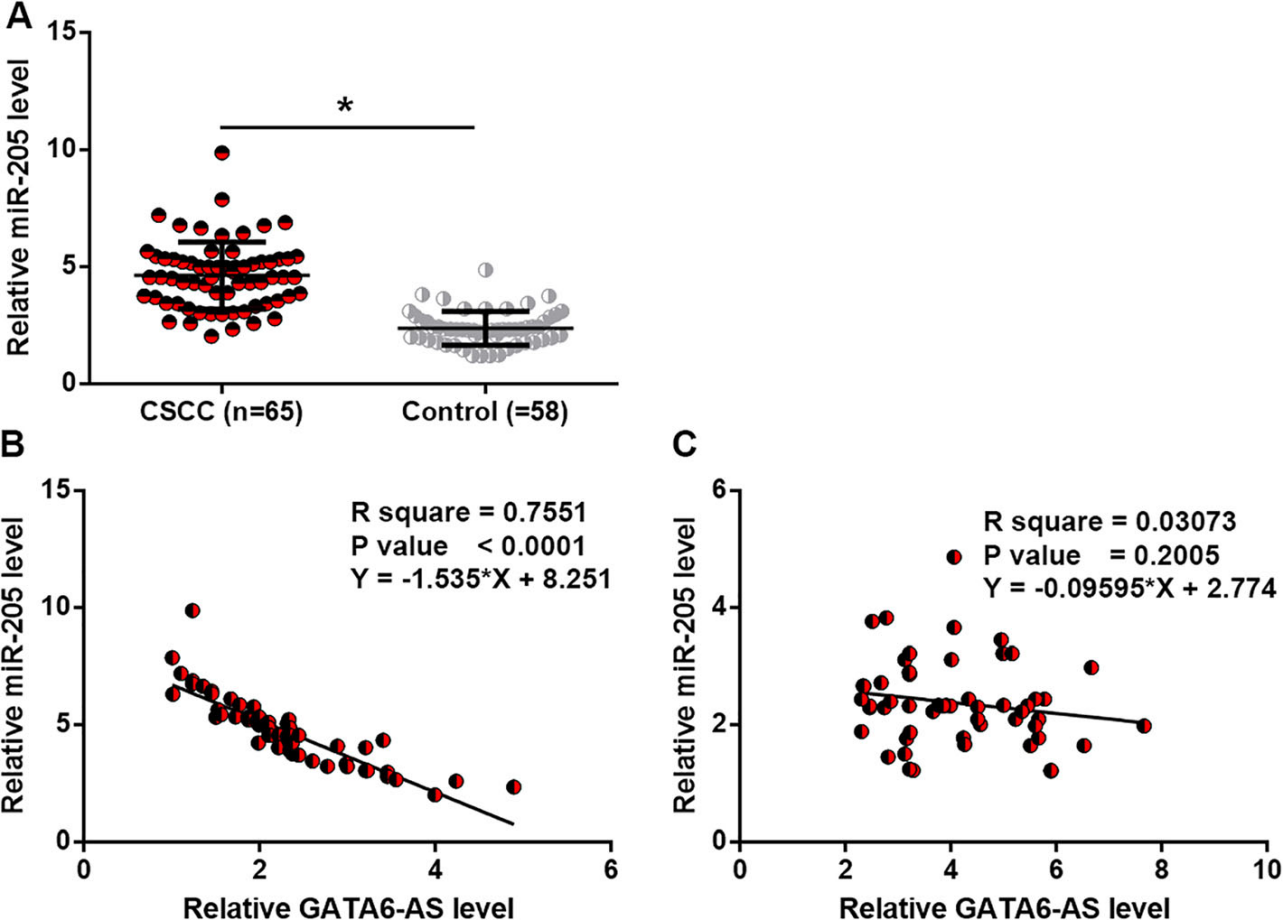
Plasma miR-205 was upregulated in CSCC patients and inversely correlated with GATA6-AS. RT-qPCR data showed that plasma levels of miR-205 were higher in CSCC patients than that in healthy females (**a**) and inversely correlated with GATA6-AS in CSCC patients (**b**), but not in healthy controls (**c**), (*, *p* < 0.05)

RT-qPCR was also performed to detect miR-205 in plasma of 65 CSCC patients and 58 healthy females. Compared with healthy females, plasma levels of miR-205 were significantly higher in CSCC patients (Fig. 3a, *p* < 0.05). Linear regression was performed to analyze the correlation between plasma levels of miR-205 and GATA6-AS. It was observed that miR-205 and GATA6-AS expression in plasma were inversely correlated in CSCC patients (Fig. 3b), but not in healthy controls (Fig. 3c).

### GATA6-AS over-expression resulted in the down-regulation of miR-205 expression in CSCC cells

**Fig. 4:**
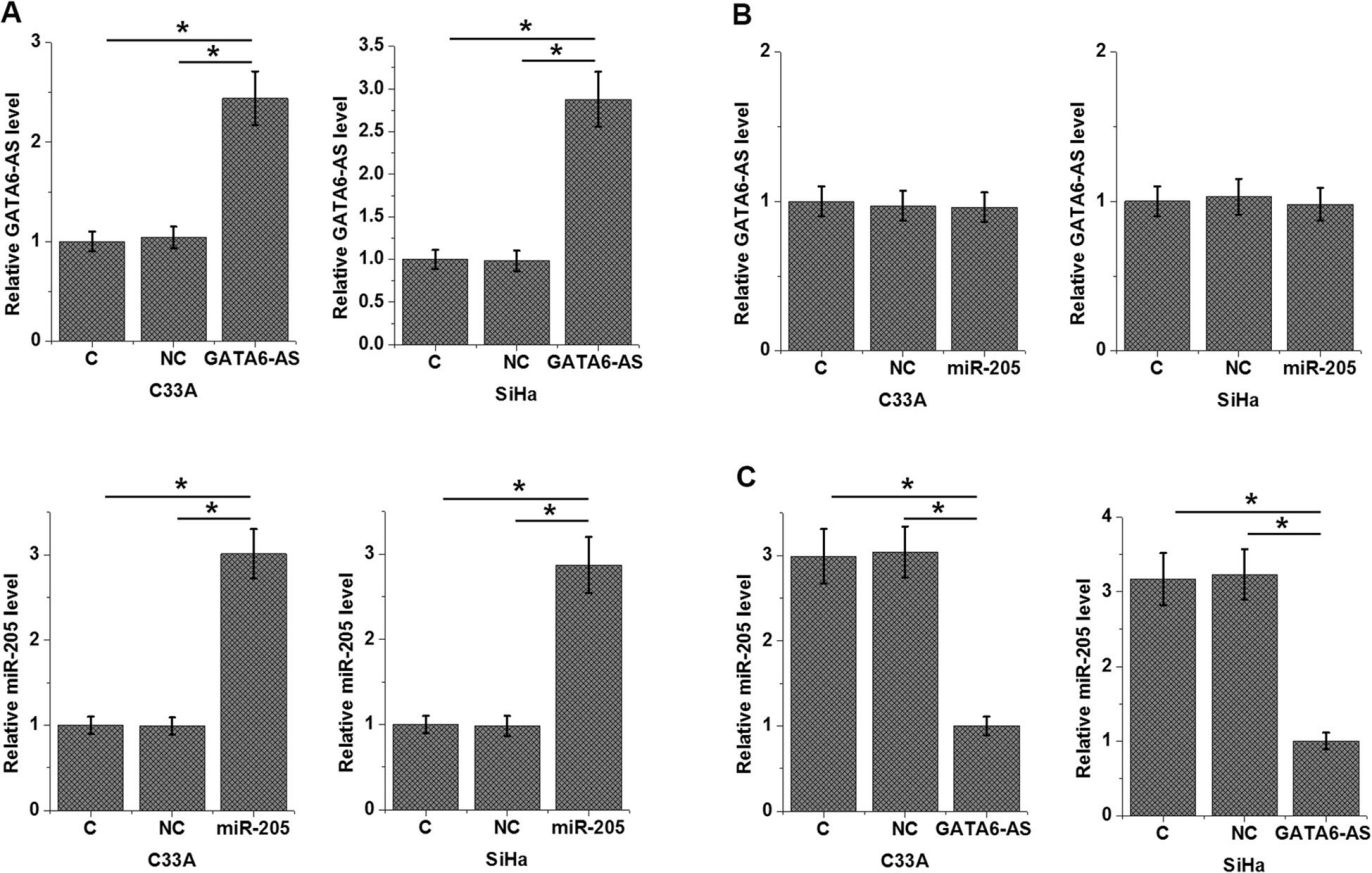
GATA6-AS over-expression resulted in the down-regulation of miR-205 in CSCC cells. C33A and SiHa cells showed significantly upregulated miR-205 and GATA6-AS at 24 h after transfection (**a**), over-expression of miR-205 did not significantly affect the expression of GATA6-AS in CSCC cells (**b**), while GATA6-AS over-expression mediated the down-regulation of miR-205 (**c**), (*, *p* < 0.05)

The inverse correlation between miR-205 and GATA6-AS is strong evidence of the existence of interaction between these two factors. To further test it, both of C33A and SiHa cells were transfected with miR-205 mimics and GATA6-AS expression vectors. As shown in Fig. 4a, C33A and SiHa cells showed significantly upregulated miR-205 and GATA6-AS expression at 24 h after transfection compared with control (C) and negative control (NC) cells (*p* < 0.05). Over-expression of miR-205 did not significantly affect the expression of GATA6-AS in CSCC cells (Fig. 4b), while GATA6-AS over-expression mediated the down-regulation of miR-205 (Fig. 4c, *p* < 0.05).

### GATA6-AS regulated CSCC cell proliferation and apoptosis through miR-205

**Fig. 5:**
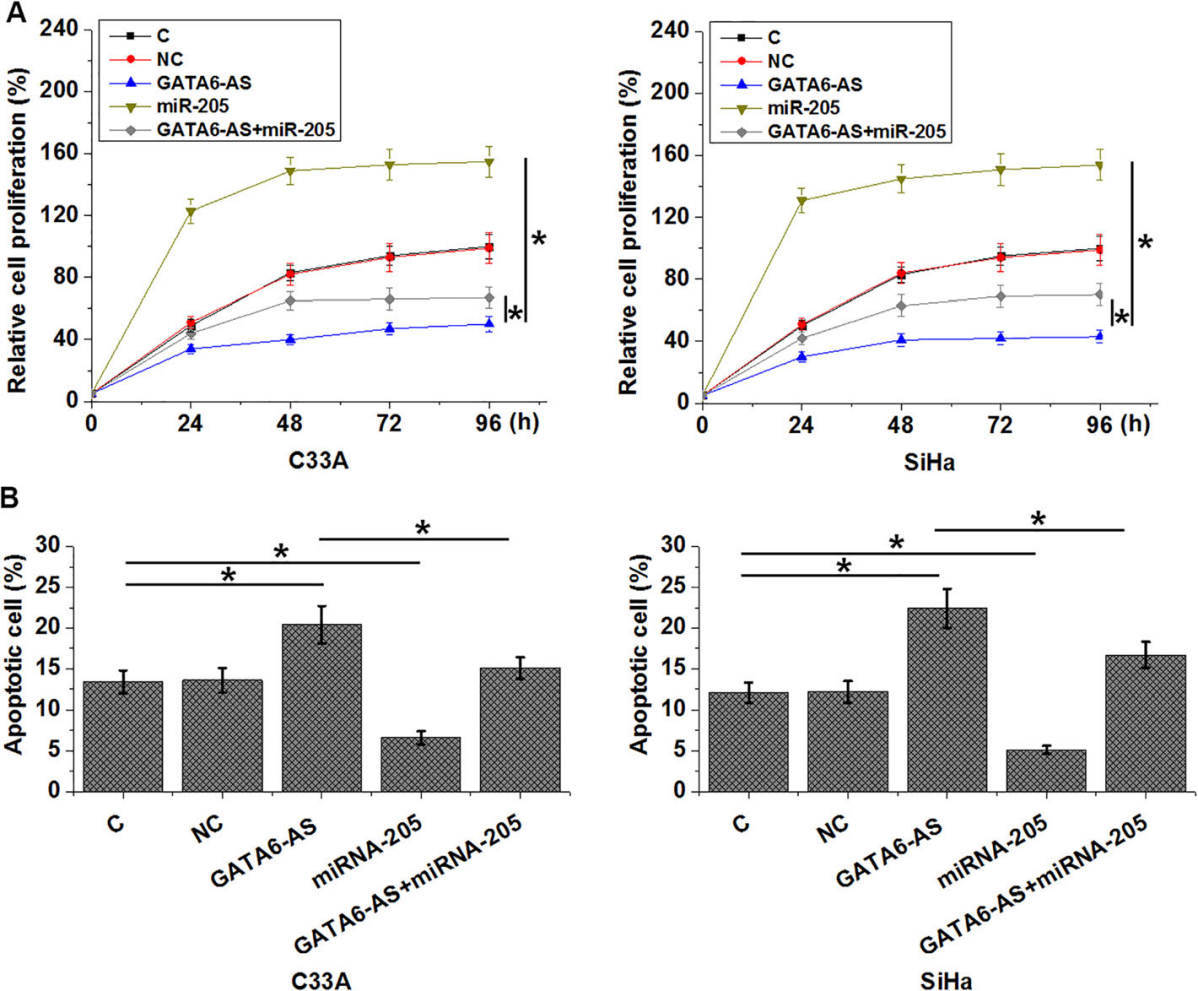
GATA6-AS regulated CSCC cell proliferation and apoptosis through miR-205. GATA6-AS over-expression inhibited proliferation (**a**) and promoted apoptosis (**b**) of CSCC cells. Over-expression of miR-205 played opposite roles and attenuated the effects of GATA6-AS over-expression on CSCC cells

Compared with control (C) and negative control (NC) groups, cells with GATA6-AS over-expression showed significantly inhibited proliferation (Fig. 5a) and promoted apoptosis (Fig. 5b) of CSCC cells (*p* < 0.05). In contrast, cells with miR-205 over-expression showed significantly promoted proliferation (Fig. 5a) and inhibited apoptosis (Fig. 5b) of CSCC cells (*p* < 0.05). In addition, over-expression of miR-205 attenuated the effects of GATA6-AS over-expression on CSCC cells (*p* < 0.05).

## Discussion

The regulatory role of GATA6-AS in endothelial cell growth indicates its involvement in many types of human diseases, such as CSCC. Our study first investigated the involvement of GATA6-AS in CSCC and proved that GATA6-AS may have tumor-suppressive function in CSCC, and this function was at least partially mediated through the interaction with miR-205.

The role of miR-205 has been characterized in many types of cancer [13–15]. Interestingly, it was observed that miR-205 might play different roles in different types of human cancer. During the development of prostate cancer, miR-205 inhibited tumor development by down-regulating the expression of protein kinase Cε [14]. In contrast, miR-205 in non–small cell lung cancer targeted PHLPP2 and PTEN and enhanced AKT signaling, resulting in cancer development [15]. In addition, Xie, et al. showed that over-expression of miR-205 in cervical cancer was closely correlated with the promoted cancer cell proliferation and migration, indicating its oncogenic role in this disease [15]. Consistent with this study, our study also proved that circulating plasma miR-205 was upregulated in CSCC patients, and over-expression of miR-205 promoted CSCC cell proliferation and inhibited CSCC cell apoptosis. Therefore, our study confirmed the oncogenic role of miR-205 in CSCC.

GATA6-AS epigenetically regulates endothelial gene expression to affect endothelial cell growth, and the altered growth of the endothelial cell is involved in CSCC [11]. Our study first reported that GATA6-AS had tumor-suppressive roles in CSCC. In addition, we found that GATA6-AS might achieve its regulatory roles of cancer cell proliferation and invasion by serving as the upstream inhibitor of miR-205. It is known that lncRNAs may down-regulate miRNAs by serving as miRNA sponge [16, 17]. However, we failed to find promising target site of miR-205 on GATA6-AS. In addition, plasma levels of miR-205 and GATA6-AS were not significantly correlated in healthy controls. Therefore, the interaction between miR-205 and GATA6-AS may be indirect, indicating the potential existence of pathological mediators between miR-205 and GATA6-AS.

## Conclusion

In conclusion, GATA6-AS has tumor-suppressive roles in CSCC by down-regulating miR-205 expression.

## Data Availability

The analyzed data generated during the study are available from the corresponding author on reasonable request.

## Abbreviations

HPV: Human papillomaviruses
CSCC: Cervical squamous cell carcinoma
HPV positive: SiHa
ncRNAs: Non-coding RNAs
lncRNAs: Long > 200 nt) ncRNAs
FBS: Fetal bovine serum
PI: Propidium iodide
C: Control
NC: Negative control
